# Interleukin-17D Promotes Pathogenicity During Infection by Suppressing CD8 T Cell Activity

**DOI:** 10.3389/fimmu.2019.01172

**Published:** 2019-06-06

**Authors:** Younghee Lee, Jelita Clinton, Chengfang Yao, Seon Hee Chang

**Affiliations:** Department of Immunology, The University of Texas MD Anderson Cancer Center, Houston, TX, United States

**Keywords:** IL-17D, listeria, flu virus, CD8, dendritic cells

## Abstract

Interleukin-17D (IL-17D) belongs to the IL-17 family of cytokines. While the members of the IL-17 family have been implicated in inflammation and host defense, the function of IL-17D remains unclear. Here, we showed that the lack of IL-17D expression confers protection against *Listeria* infection. A deficiency in IL-17D also resulted in less weight loss with reduced pathogen burden during influenza A virus infection. During infection, the loss of IL-17D resulted in compromised CD8 T cell activity. CD8 T cell depletion in IL-17D-deficient mice restored the bacterial burden to a level similar to that found in WT mice. Similarly, IL-17D-deficient mice in a RAG-deficient background had no difference in bacterial and viral burden compared to WT mice. IL-17D controlled CD8 T cell activity in part by suppressing the function of dendritic cells. We found that IL-17D from the non-hematopoietic compartment regulates protective immunity during infection. Together, our data led to the identification of IL-17D as a critical cytokine during intracellular bacteria and virus infection that suppresses the activity of CD8 T cells by regulating dendritic cells.

## Introduction

The IL-17 family of cytokines, which was identified relatively recently, is closely linked to host defense and immune response (1–4). Each cytokine in this family plays a unique and non-redundant function in infection, autoimmunity, and cancer. IL-17A, the first identified member of the IL-17 cytokine family, has the critical function of promoting neutrophil recruitment during bacterial and fungal infection. However, sustained production of IL-17A and its homologous protein, IL-17F, which are produced by adaptive immune CD4 T cells that are recruited to the inflamed lesions, promotes tissue inflammation. Recently, we demonstrated that IL-17C is necessary to promote autoimmunity and maintain mucosal barrier integrity (5, 6). IL-17C also promotes epithelial inflammation (7) and host defense during colitogenic enterobacterial infection (8). IL-17E (IL-25), which potentiates allergic inflammation, mediates a strong host defense during parasitic and fungal infection (9–11). Therefore, accumulating evidence indicates that members of the IL-17 family of cytokines are required for host defense during infection.

IL-17D is a novel cytokine in the IL-17 family of cytokines that has not been extensively investigated. A recent report indicated that it is highly secreted by fibrosarcoma tumor cells; in addition, ectopic expression of IL-17D in tumor cells recruits natural killer cells via the CCL2 production of endothelial cells (12). IL-17D is positively regulated by Nrf2 (13), and its expression is suppressed in advanced cancer (12). However, the role of endogenous IL-17D in immunity remains poorly understood.

To comprehensively understand the roles of IL-17D in inflammation and host defense, we examined IL-17D-deficient animals in steady state and during inflammation. While IL-17D was redundant in several acute and chronic inflammation models, IL-17D-deficient mice were resistant to *Listeria* infection with an increased cytotoxic CD8 T cell response compared to WT mice. A reduced pathogen burden in IL-17D-deficient mice was also observed after influenza A virus infection. IL-17D suppressed the activity of dendritic cells (DCs) isolated from the mice infected with *Listeria*, which resulted in the suppression of CD8 T cells. We further demonstrated that IL-17D from the non-hematopoietic compartment exercises a pathogenic role and IL-17D expression is repressed in the infected liver tissue. These results suggest that IL-17D is a unique member of the IL-17 family that regulates protective immunity during intracellular bacteria and virus infection via regulating CD8 T cells.

## Materials and Methods

### Mice

*Il17d*-targeted mice, *Il17d* ^tm1Lex^, were generated at Genentech and Lexicon Pharmaceuticals by targeting exon 3 of *Il17d* gene by homologous recombination. This strain was purchased from the MMRRC facility (032380-UCD-SPERM) as cryo-preserved spermatozoa, and *in vitro* fertilization was performed at The University of Texas MD Anderson Cancer Center Genetically Engineered Mouse Facility. Heterozygous (*Il17d* ^+/−^) mice were bred to generate age-matched WT and homozygous *Il17d* ^−/−^ mice for experiments. *Rag1*^−/−^ mice (stock number: 002216) and OT-1 TCR Tg mice (stock number: 002216) were obtained from the Jackson Laboratory. Mice were maintained in a specific pathogen-free facility, and all animal experiments were conducted in accordance with protocols approved by the Institutional Animal Care and Use Committee of MD Anderson Cancer Center.

### Bacteria Culture and Infection

An erythromycin-resistant strain of LM-OVA was grown in brain heart infusion media supplemented with 5 μg/ml erythromycin (14, 15). The bacteria were intravenously injected into WT or *Il17d*^−/−^ mice (1 × 10^4^ /mouse), and the spleen and liver were analyzed 3 or 7 days after infection. Bacterial burdens were determined by counting the number of CFUs (colony forming units) in the serially diluted homogenized spleen and liver using a BHI agar plate. Total livers were resuspended in Trizol for the mRNA expression analysis. Splenocytes or liver mononuclear cells were stimulated with SIINFEKL peptide or LLO peptide overnight for intracellular cytokine staining. To deplete CD4 or CD8 T cells, 100 μg of anti-CD4 mAb (GK1.5) or anti-CD8 mAb (53–6.7) were injected into mice via an intraperitoneal route on days −3 and −1.

For the influenza A virus infection model, WT or *Il17d*^−/−^ mice were infected with 13 plaque-forming units (PFUs) of influenza virus A H1N1 strain A/Puerto Rico/8/34 (PR8) (Charles River Laboratories), and their body weight was monitored daily. The expression of viral hemagglutinin (HA) in the lungs of infected mice was determined by RT-PCR. PCR primers used were as follows: influenza HA primers (forward: 5′-GGTGTTCATCACCCGTCTAACAT-3′, reverse: 5′-TGTTTGACACTTCGCGTCACAT-3′) (16).

### Tumor Injection

WT or *Il17d*^−/−^ mice were subcutaneously injected with 1 × 10^6^ E.G7 OVA cells (T cell lymphomas), and tumor size was monitored using a caliper. Mice were killed when the tumor diameters reached 15 mm. Tumor volume was calculated using the ellipse formula, length/2 × width/2 × pi. For the melanoma model, WT and *Il17d*^−/−^ mice were inoculated intravenously with 1 × 10^5^ B16 OVA; 2 weeks later, they were euthanized to evaluate tumor foci numbers in the lungs (17). OVA^257−264^ (SIINFEKL) peptide bound to H-2Kb antibody was obtained from ProImmune.

### Quantitative Real-Time PCR

To analyze mRNA expression, tissue samples or cells were homogenized in TRIzol (Invitrogen). RT-PCR was performed using IQ SYBR Green on a CFX96 instrument (Bio-Rad). The primers used included *Il17d*: forward (F), 5′-GGGCGTACAGGATTTCCTAC-3′ and reverse (R), 5′-AGAGAAGACGGGTGTGCTG-3′; *Il6*: (F) 5′-ACCAGAGGAAATTTTCAATAGGC-3′; (R) 5′-TGATGCACTTGCAGAAAACA-3′y; *Ifng*: (F) 5′y-GATGCATTCATGAGTATTGCCAAGT-3′; (R) 5′-GTGGACCACTCGGATGAGCTC; *Tnf*α: (F) 5′y-AATGGCCTCCCTCTCATCAGT-3′y; (R) 5′-GCTACAGGCTTGTCACTCGAATT-3′y; *Il23*: (F) 5′-CCAGCGGGACATATGAATCT-3′; (R) 4′-AGGCTCCCCTTTGAAGATGT-3′y; *Il12a*: (F) 5′-GAGGACTTGAAGATGTACCAG-3′yy; (R) 5′yy-CTATCTGTGTGAGGAGGGC-3′; *Il12b*: (F) 5′yy-GACCCTGCCCATTGAACTGGC-3′; (R) 5′yy-CAACGTTGCATCCTAGGATCG-3′; *Ccl2*: (F) 5′-CTCAGCCAGATGCAGTTAACGCCC-3′; (R) 5′-GGTGCTGAAGACCTTAGGGCAGAT-3; *Cxcl1*: (F) 5′-CGCTTCTCTGTGCAGCGCTGCTGCT-3′; (R) 5′-AAGCCTCGCGACCATTCTTGAGTG-3′; *Cxcl2*: (F) 5′-CGCCCAGACAGAAGTCATAG-3′; and (R) 5′-TCCTCCTTTCCAGGTCAGTTA-3′.

### ELISA

WT was i.v. infected with 1 × 10^4^ LM-OVA and liver and spleens were isolated on day 7. *Il17d*^−/−^ mice were used as a negative control. Livers were homogenized with protein extract buffer (20 mM HEPES, 250 mM Nacl, 0.5% NP-40, 1 mM EDTA, 20 mM β-glycerophosphate, protease inhibitor cocktails) and the supernatants were collected. IL-17D protein concentration was measured using mouse IL-17D Duoset ELISA (R&D systems, DY2274).

### Antibodies and Flow Cytometry

The following fluorescent dye-conjugated antibodies to mouse proteins were purchased from BD Biosciences: CD4 (GK1.5), CD8 (53-6.7), CD11b (M1/70), Gr1 (1A8), NK1.1 (PK136), IFNγ (XMG1.2), and TNFα (MP6-XT22). Antibodies against Granzyme B (GB11), CD25 (PC61), CD44 (IM7), and CD62L (MEL-14) were purchased from Biolegend. IL-17A (eBio17B7) was purchased from eBioscience.

### OT I T Cell Proliferation Assay

DCs were isolated from the spleens and lymph nodes of LM-OVA-infected WT mice using anti-CD11c magnetic beads and automacs. They were then incubated with 20 μg/ml OVA solution for 1 h. OT-I CD8 T cells were isolated using anti-CD8 magnetic beads and labeled with 10 mM CTV (CellTrace Violet, Thermo Fisher Scientific). DCs and CTV-labeled CD8 T cells were co-cultured with IL-17D (200-0 ng/ml, 2274-ML, R&D Systems). After 3 days, the cells were intracellularly stained with PE-conjugated anti-Vα2 Ab before being permeabilized and further stained with APC-conjugated anti-IFNγ Ab. Heat inactivated IL-17D was prepared by heating at 56°C for 30 min.

### Generation of BM Chimeras

Recipient WT or *Il17d*^−/−^ mice (6–8 weeks) were irradiated with 750 rad using a cesium irradiator and injected intravenously with 5 × 10^6^ BM cells derived from the femurs of the WT or *Il17d*^−/−^ donors. After 6–8 weeks, reconstituted mice were infected using LM-OVA or injected with EG.7 OVA tumor cells.

### LPS-Induced Septic Shock

WT or *Il17d*^−/−^ mice were injected intraperitoneally with LPS (12 mg/kg and 5 mg/kg). Survival was monitored up to 72 h. Serums were collected in different time points and subjected for ELISA.

### KLH Immunization

WT or *Il17d*^−/−^ mice were subcutaneously immunized at the tail base with 50 μl of KLH (0.5 mg/ml), emulsified in complete Freund's adjuvant (CFA). Seven days later, the spleens and draining lymph nodes of immunized mice were analyzed.

### EAE Immunization

WT or *Il17d*^−/−^ mice were immunized with 300 μg of MOG peptide (amino acids 35–55) emulsified in CFA, followed by an intraperitoneal injection of pertussis toxin (100 μl) on days 0 and 2 (5). Disease scores were assigned on a scale of 0–5, as follows: 0, none; 1, limp tail or waddling gait with tail tonicity; 2, wobbly gait; 3, hindlimb paralysis; 4, hindlimb and forelimb paralysis.

### OVA-Induced Allergic Lung Inflammation

WT or *Il17d*^−/−^ mice were immunized intraperitoneal with 50 μg of OVA (Albumin from chicken egg, grade VII, Sigma) emulsified in alum on days 0 and 14, followed by intranasal administration of 50 μg of OVA on days 14, 25, and 26, as described previously (18). After 28 days, BAL fluid was collected for leukocyte infiltration by Diff-Quik histological staining or IL-4, IL-5, and IL-13 determination by ELISA.

### Statistical Analysis

Statistical comparisons were performed using a two-tailed unpaired Student's *t*-test, as indicated. *P*-values < 0.05 were considered statistically significant.

## Results

### IL-17D Is Redundant in Acute and Chronic Inflammation Animal Models

**Figure 1:**
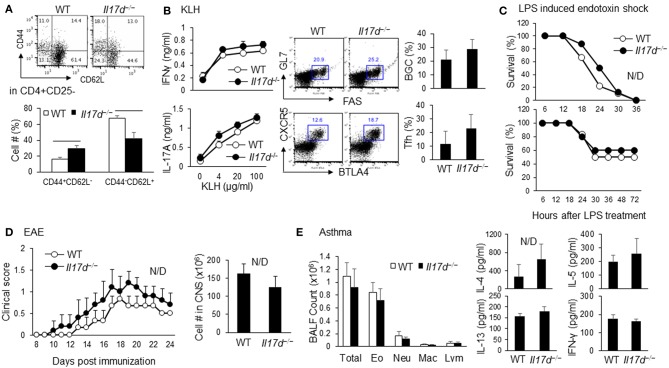
Characterization of *Il17d*^−/−^ mice in steady state and acute and chronic inflammation. **(A)** WT (*n* = 3) or *Il17d*^−/−^ (*n* = 4) were analyzed at 14–16 weeks old. Effector/memory T cells (CD44^hi^CD62^lo^) and naïve T cells (CD44^lo^CD62L^hi^) in CD4+CD25– T cells were analyzed by FACS. **(B)** Spleens were re-stimulated with KLH for 3 days and subjected to IFNγ and IL-17 ELISA. Follicular helper T cells (CXCR5^+^BTLA^+^ in CD4^+^ T cells) and Germinal center B cells (GL7^+^FAS^+^ on B cells) were analyzed in splenocytes from WT (*n* = 5) or *Il17d*^−/−^ mice (*n* = 4) 7 days after KLH/CFA immunization. **(C)** WT and *Il17d*^−/−^ mice were injected intraperitoneal with 12 mg/kg LPS (Top, WT *n* = 9; *Il17d*^−/−^ *n* = 8) and 5 mg/kg LPS (Bottom, WT *n* = 6; *Il17d*^−/−^ *n* = 5). Survival was monitored up to 72 h. **(D)** Clinical scores of WT (*n* = 5) or *Il17d*^−/−^ mice (*n* = 3) after EAE induction. Mice were immunized with MOG/CFA and injected with pertussis toxin; disease progression was monitored daily. Numbers of infiltrated cells in the CNS of WT or *Il17d*^−/−^ mice on day 24. **(E)** Total cell counts and the presence of Th2 cytokines (IL-4, IL-5, and IL-13) in the BAL fluid of WT or *Il17d*^−/−^ mice immunized with OVA in alum on days 0 and 14, followed by intranasal challenge with OVA on days 14, 25, and 26. Data are from two to three experiments.

The IL-17D homozygous knockout (*Il17d*^−/−^) mice were born in the expected Mendelian ratios and had similar survival rates to those of the wild-type (WT) control mice. *Il17d*^−/−^ mice had no obvious abnormalities at a young age (5–8 weeks; data not shown) and had a moderate increase in memory CD44^hi^CD62^lo^ cells at an older age (14–16 weeks; Figure 1A). We used the protein antigen-challenged model, Keyhole Limpet Hemocyanin (KLH) immunization, and found that *Il17d*^−/−^ mice did not have an increased antigen-specific IFNγ response compared to WT mice, despite more activated CD4 T cells in lymphoid organs in steady state (Figure 1B). Since other IL-17 family members are critical in the pathogenesis of various acute or chronic inflammatory conditions, we initially challenged *Il17d*^−/−^ mice in these animal models of inflammation. IL-17D was dispensable in several inflammation settings, lipopolysaccharide (LPS)-induced endotoxin shock, experimental autoimmune encephalomyelitis (EAE), and allergic lung inflammation (Figures 1C–E, Supplementary Figure 1).

### IL-17D Promotes Chronicity of LM-OVA Infection in Mice

**Figure 2:**
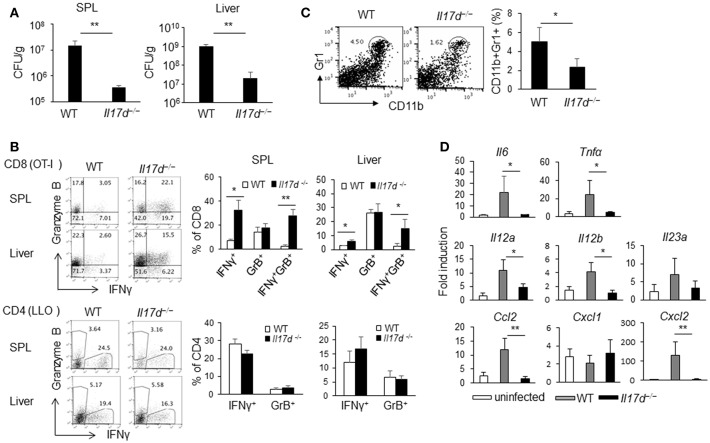
IL-17D promotes chronicity of LM-OVA infection in mice. **(A)** WT (*n* = 5) or *Il17d*^−/−^ mice (*n* = 5) were intravenously infected with 1 × 10^4^ LM-OVA on day 0, and the mice were analyzed on day 7. Livers and spleens were collected, homogenized, and counted for bacterial burden by serial dilution on BHI agar after overnight incubation. **(B)** CD4 or CD8 T cells isolated from spleen and liver and re-stimulated with specific peptide (1 μg/ml of LLO peptide for CD4, 1 μg/ml of OT1 peptide for CD8) for overnight. IFNγ and granzyme B production in CD4 and CD8 T cells analyzed by intracellular staining. **(C)** CD11b^+^Gr1^+^ cells in the spleen were stained by FACS. **(D)** Molecular analysis by RT-PCR in the liver. Data are representative of at least five independent experiments. _*_p ≤ 0.05, _**_p ≤ 0.01.

To determine whether IL-17D plays a role in the host defense during acute infection like other IL-17 family members (IL-17A, IL-17F, IL-17C, and IL-25), we challenged *Il17d*^−/−^ mice with lethal doses of *Listeria monocytogenes*-expressing ovalbumin (LM-OVA), an intracellular pathogen. We observed that *Il17d*^−/−^ mice exhibited less bacterial burden than did WT mice (spleen: 14.7 × 10^6^ colony-forming units [CFUs]/g WT and 0.36 × 10^6^ CFUs/g KO, *p* = 0.003; liver: 10.3 × 10^8^ CFUs/g WT and 0.2 × 10^8^ CFUs/g KO, *p* = 0.0001) (Figure 2A).

A cellular analysis on day 7 after infection indicated that there was an enhanced antigen-specific CD8 (OT1 peptide) immune response (Figure 2B) in *Il17d*^−/−^ mice while CD4 (LLO peptide) response was similar between WT and *Il17d*^−/−^ mice. Apparently, *Il17d*^−/−^ mice did not exhibit any enhanced innate immunity on day 3 (data not shown), suggesting that IL-17D mediates the pathogenicity of LM-OVA by means other than through suppression of the innate immune response. Bacterial burden between WT and *Il17d*^−/−^ mice was also similar on day 3 (Supplementary Figure 2A). IL-17D was dispensable during LPS-induced endotoxin shock (Figure 1C), supporting the redundant role of IL-17D in innate immunity. The frequency of Gr1^+^CD11b^+^ myeloid cells were reduced in *Il17d*^−/−^ mice (Figure 2C). Reduced number of neutrophils in the spleen of *Il17d*^−/−^ mice could be a consequence of reduced bacterial burden on day 7. We also examined inflammatory molecules in the infected liver. Expression of several pro-inflammatory molecules, such as *Il6, Tnf*α*, Cxcl2, Ccl2, Il12a, Il12b* were reduced while *Cxcl1* and *Il23a* remained similar between WT and *Il17d*^−/−^ mice (Figure 2D). We also examined the susceptibility to listeria with another strain which does not express OVA, *L. monocytogenes* ATCC strain 13932. Similar to LM-OVA, livers of *Il17d*^−/−^ mice had reduced bacteria burden in comparison to those of WT mice on day 7 (Supplementary Figure 2B). The survival of infected animals was monitored after *L. monocytogenes* ATCC strain 13932 infection. WT mice began to die on day 5, with no survival (0/9) by day 7, compared with 50% survival (4/8) for *Il17d*^−/−^ mice (*p* = 0.016) (Supplementary Figure 2C).

### Severity of Influenza a Virus Infection Is Reduced in *Il17d*^−/−^ Mice

**Figure 3:**
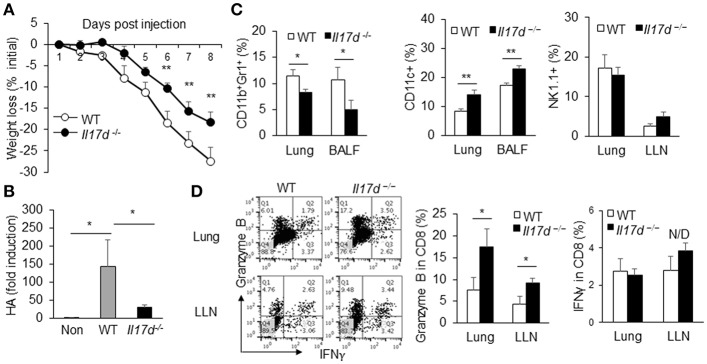
IL-17D promotes pathogen burden of influenza A virus infection in mice. **(A)** WT (*n* = 5) and *Il17d*^−/−^ mice (*n* = 4) were intranasal administrated with 13 PFUs of influenza A virus, and changes in body weight of WT and KO mice were monitored daily up to day 8. **(B)** Lung viral load was assessed via by qPCR of influenza A HA RNA. **(C)** BAL fluid cells and lung mononuclear cells were isolated and analyzed. CD11b^+^Gr1^+^ and CD11c cells from BAL fluid and lung mononuclear cells were stained by FACS. NK cells were examined in lung mononuclear cells and draining lymph nodes by FACS. **(D)** Production of granzyme B and IFNγ in CD8 T cells in the lung and lung-draining lymph nodes by intracellular staining on day 9. The CD8 T cells were re-stimulated with PMA and ionomycin for 5 h before the staining. Data are representative of at least two or three independent experiments. _*_p ≤ 0.05, _**_p ≤ 0.01.

To determine whether IL-17D generally promotes pathogenicity during infection, we challenged WT and *Il17d*^−/−^ mice with PR8 influenza A virus. Of note, we also observed that *Il17d*^−/−^ mice were partially resistant to influenza infection (Figure 3A) on the basis of a weight loss difference, suggesting that IL-17D is a pathogenic molecule during acute virus infection. This result was confirmed by RT-PCR analyses that examined the abundance of viral hemagglutinin (HA) mRNA in lung homogenates of PR8-infected mice. As shown in Figure 3B, HA expression was significantly lower in the lungs of PR8-infected *Il17d*^−/−^ mice compared to PR8-infected WT mice on post-infection day 8. The frequency of CD11b^+^Gr1^+^ myeloid cells was reduced and the number of CD11c^+^ DCs was increased in both lung and bronchoalveolar lavage (BAL) fluid cells in *Il17d*^−/−^ mice (Figure 3C). The frequency of NK cells remained similar in lung and draining lymph nodes (Figure 3C). CD8 T cells in the draining lymph nodes and lungs of *Il17d*^−/−^ mice expressed higher levels of granzyme B than did those of WT mice (Figure 3D). IFNγ production from CD8 T cells remained comparable between WT and *Il17d*^−/−^ mice. These results indicate that IL-17D is a novel cytokine that is harmful to the host in the context of acute infection by suppressing the adaptive immune response and enhancing inflammation.

### IL-17D Reduces Cytotoxic T Lymphocyte Response During LM-OVA Infection in Mice

**Figure 4:**
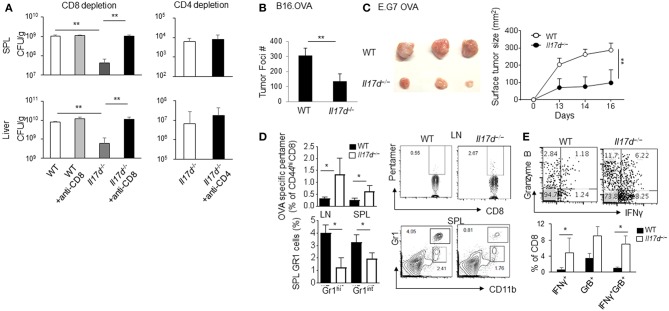
IL-17D promotes chronicity of LM-OVA infection by suppressing CD8 T cells, not CD4 T cells. **(A)** WT (*n* = 4) or *Il17d*^−/−^ mice (*n* = 4) were intravenously infected with 1 × 10^4^ LM-OVA. Anti-CD8 (536.7) and anti-CD4 (GK1.5) mAb were injected 1 and 3 days before the bacterial challenge. Bacterial burden was counted on day 7. **(B)** B16 OVA melanoma cells were intravenously injected into WT (*n* = 5) or *Il17d*^−/−^ (*n* = 4) mice. After 14 days, mice were killed, and the tumor burdens were counted. **(C)** E.G7 OVA lymphoma cells were subcutaneously injected into WT (*n* = 3) or *Il17d*^−/−^ (*n* = 3) mice. After 16 days, mice were killed, and their tumor size and tumor weight were measured. **(D)** OVA-specific pentamer-positive cells in CD8 T cells in spleens and lymph nodes. Gr1^hi^ CD11b+ and Gr1^int^ CD11b+ gated cell percentages in the spleen and lymph nodes. **(E)** Production of granzyme B and IFNγ in CD8 T cells in tumor infiltrating lymphocytes by intracellular staining. Data are representative of at least two or three independent experiments. _*_p ≤ 0.05, _**_p ≤ 0.01.

To better understand how IL-17D promotes pathogenicity in infection, we focused on LM-OVA infection, as it is relatively straightforward to dissect its cellular mechanism. Since IL-17D seemed to modulate the adaptive immune response, we depleted CD8 T cells in *Il17d*^−/−^ mice during LM-OVA infection. CD8 depletion in *Il17d*^−/−^ mice restored bacterial burden in *Il17d*^−/−^ animals (Figure 4A). CD4 depletion, however, did not reverse the bacterial burden, indicating that IL-17D exerts a pathogenic function during *Listeria* infection by repressing CD8 T cell activity. Since CD8 T cells are a key mediator of anti-tumor immunity, we examined whether *Il17d*^−/−^ mice exhibited enhanced CD8 T cell activity upon tumor challenge. We employed a well-established ectopic tumor transplantation model in which CD8 T cells are critical for controlling the tumor. We found that *Il17d*^−/−^ mice were resistant to tumor growth upon B16 OVA melanoma and E.G7 OVA lymphoma challenge (Figures 4B,C). An analysis of tumor-infiltrated cells and lymphoid organs revealed the presence of enhanced cytotoxic CD8 T cell activity and reduced CD11b^+^Gr1^+^ myeloid cells in *Il17d*^−/−^ mice upon tumor challenge (Figures 4D,E). These results support the notion that CD8 T cells are key cell types that are regulated by IL-17D in the context of infection and tumor.

### IL-17D Suppresses DC Activation in the Absence of Adaptive Immunity

**Figure 5:**
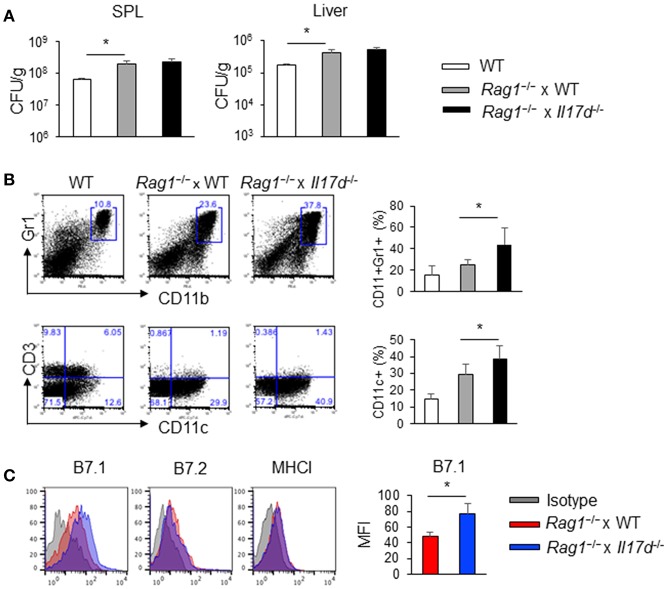
IL-17D suppresses DC activation in *Rag1*^−/−^ mice. WT or *Rag1*^−/−^ or *Rag1*^−/−^ x *Il17d*^−/−^ mice were intravenously infected with 1 × 10^4^ LM-OVA on day 0. Mice were sacrificed and analyzed on day 7. **(A)** Bacterial burden in the spleen and liver. **(B)** Cellularity of CD11b^+^Gr1^+^ cells and CD11c^+^ cells in the spleen by FACS staining. **(C)** DC activation markers (CD80, CD86, and MHC I) were stained by FACS. Data are representative of three independent experiments. _*_p ≤ 0.05.

To demonstrate that the anti-*Listeria* function by IL-17D is mediated by the suppression of CD8 T cells using another experimental system, we crossed *Il17d*^−/−^ mice with recombinase-activating gene-1 (RAG-1)-deficient mice, which have developmental deficiencies in T cells, B cells, γ-δ T cells, and NKT cells. In the absence of an adaptive immune response, *Rag1*^−/−^ and *Rag1*^−/−^ x *Il17d*^−/−^ mice exhibited similar levels of bacterial burden (Figure 5A). Similarly, we observed that *Rag1*^−/−^ and *Rag1*^−/−^ x *Il17d*^−/−^ mice exhibited comparable weight loss and HA expression after infection with influenza A virus with similar viral load in lung (Supplementary Figures 3A,B). When infected with LM-OVA, frequency of DCs (CD11c^+^) and neutrophils (CD11b^+^Gr1^+^) were higher in *Rag1*^−/−^ x *Il17d*^−/−^ mice than in *Rag1*^−/−^ mice (Figure 5B). DCs from *Rag1*^−/−^ x *Il17d*^−/−^ mice expressed an increased level of costimulatory molecule B7.1, while the levels of B7.2 and MHC I were similar between *Rag1*^−/−^ and *Rag1*^−/−^ x *Il17d*^−/−^ mice (Figure 5C). However, increased numbers of innate immune cells of *Rag1*^−/−^ x *Il17d*^−/−^ mice were not sufficient to result in a difference in bacterial burden. We did not see these differences in the phenotype of innate immune cells in fully competent animals (data not shown). *Rag1*^−/−^ mice may manifest these differences in innate immune cells in the absence of T and B cells. During *Listeria* infection, CD8 T cell activity is determined by various factors, such as DCs. Therefore, this result suggests that enhanced activation of CD8 T cells in *Il17d*^−/−^ mice is due to more activated DCs.

### IL-17D Suppresses DC Activation to Reduce CD8 T Cell Activation During LM-OVA Infection in Mice

**Figure 6:**
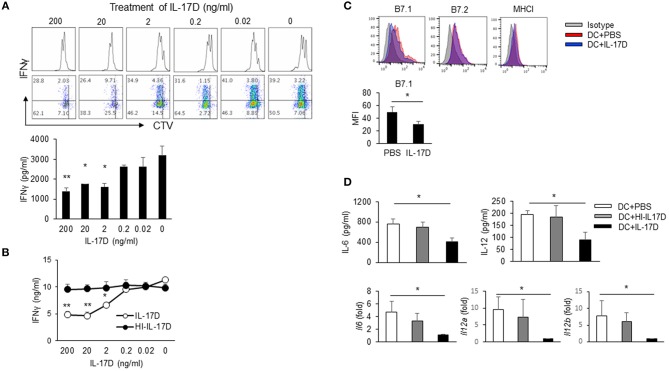
IL-17D regulates CD8 T cell proliferation by DC suppression. **(A)** DCs from LM-OVA-infected WT mice were pulsed with soluble OVA (20 μg/ml). CTV-labeled OTI CD8 T cells were co-cultured with DCs and IL-17D (200-0 ng/ml) for 3 days. Production of IFNγ was analyzed by intracellular staining and ELISA. **(B)** OT-I cells were co-cultured with DCs and IL-17D or heat-inactivated IL-17D (200-0 ng/ml) for 3 days, and IFNγ production was measured by ELISA. **(C,D)** DCs were isolated from LM-OVA-infected mice and treated with IL-17D (200 ng/ml) for 1 day. DC activation markers (CD80, CD86, and MHC I) were analyzed by flow cytometry. Cytokines (IL-6 and IL-12) were analyzed by ELISA and RT-PCR. Data are representative of three independent experiments. _*_p ≤ 0.05, _**_p ≤ 0.01.

To determine whether IL-17D suppresses CD8 T cells *ex vivo*, we set up a DC/CD8 co-culture system in which activated DCs were isolated from the spleens of LM-OVA-infected mice. DCs were cultured with CTV-labeled CD8 T cells that had been isolated from OT-1 TCR transgenic mice. IL-17D had no direct effects on the proliferation of CD8 T cells (data not shown). In the co-culture system, IL-17D suppressed the proliferation of OT-1 CD8 T cells and the IFNγ production of CD8 T cells in a concentration-dependent manner (Figure 6A). Heat-inactivated IL-17D (HI-IL-17D) did not affect cytokine production (Figure 6B). To determine whether IL-17D directly modulates DCs, we isolated DCs from the spleens of LM-OVA-infected mice and treated them with IL-17D (200 ng/ml) for 24 h. IL-17D suppressed B7.1 expression (Figure 6C) and cytokine production (IL-6 and IL-12) in DCs (Figure 6D). These results showed that IL-17D regulates CD8 proliferation and IFNγ production by suppressing DC activity.

### IL-17D Is Expressed in Non-hematopoietic Cells

**Figure 7:**
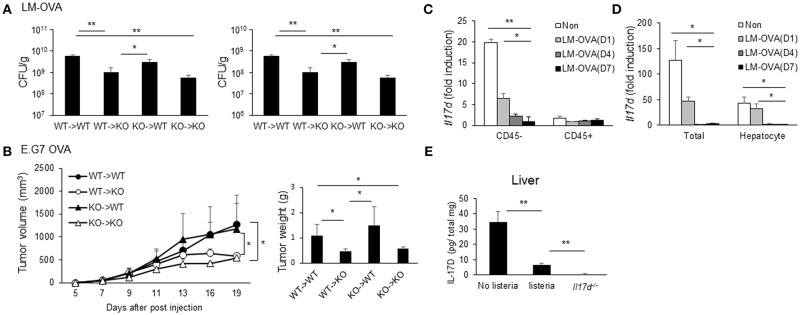
Non-hematopoietic compartments of IL-17D contribute to pathogenicity. **(A,B)** WT or *Il17d*^−/−^ BM cells were transferred to irradiated WT or *Il17d*^−/−^ mice (*n* = 3–4 per group). Eight weeks after BM reconstitution, LM-OVA **(A)** or EG.7 OVA lymphoma cells **(B)** were injected, and bacterial burden or tumor growth was measured. **(C,D)** WT mice were killed on days 1, 4, and 7 after LM-OVA infection. **(C)** Expression of IL-17D on CD45^+^ or CD45^−^ cells in the liver. **(D)** Expression of IL-17D in total liver tissues and isolated hepatocyte. **(E)** Expression of IL-17D in total liver tissues by ELISA. Data are representative of three independent experiments. _*_p ≤ 0.05, _**_p ≤ 0.01.

Since IL-17D promotes pathogenicity during infection and suppresses CD8 T cell activity, we examined the cellular compartments of IL-17D production. Irradiated WT and *Il17d*^−/−^ mice were reconstituted with bone marrow (BM) from WT or *Il17d*^−/−^ mice. After 8 weeks, the recipient mice were infected with LM-OVA and analyzed for bacterial burden (Figure 7A). Deletion of IL-17D in non-hematopoietic compartments was sufficient to replicate the phenotype observed in IL-17D-deficient animals, while the effects of IL-17D deletion in the hematopoietic compartment were minimal, suggesting that IL-17D secretion from non-hematopoietic compartments is required for the pathogenic effects of IL-17D during bacterial infection. This phenotype was also observed in the E.G7 OVA tumor model (Figure 7B).

These results indicate that a non-hematopoietic cell type is involved in the production of IL-17D. Therefore, we examined the expression of IL-17D in the liver during LM-OVA infection. Liver cells were sorted by automacs with a CD45 antibody. The CD45-negative fraction expressed a higher level of IL-17D than did the CD45-positive fraction (Figure 7C). This result is consistent with the non-hematopoietic cell type producing IL-17D from the BM chimera experiment. Next, we isolated hepatocytes in normal and infected liver tissue and found that IL-17D expression in hepatocytes progressively decreased during LM-OVA infection (Figure 7D). To ensure the reduction of IL-17D upon the infection occurs in protein level as well, we measured IL-17D using ELISA from total lysates of liver tissue before and after the infection. IL-17D protein expression was reduced after the infection similar to mRNA expression (Figure 7E). These results indicate that IL-17D is produced by non-hematopoietic cells, including hepatocytes in steady state. Furthermore, IL-17D could be repressed upon LM-OVA infection, although we cannot rule out the possibility that the IL-17D-producing cell type is less represented in the CD45-negative fraction upon infection.

Therefore, our findings indicate that IL-17D is a cytokine that is derived from non-hematopoietic cells and its expression is repressed in inflammation. IL-17D compromises host immunity during infection by suppressing DCs to modulate CD8 T cell activity.

## Discussion

IL-17 family cytokines have been implicated in the host defense against infection, autoimmunity, and inflammation. Our purpose for the current study was to investigate the biological function of IL-17D, a relatively unknown member of the IL-17 family cytokines, using IL-17D-deficient mice in various pathological settings. Since IL-17D-deficient mice were resistant to *Listeria* infection, we evaluated pathogen burden upon other infection such as influenza virus and provided unexpected insights into the role of IL-17D in compromising the host defensive function. We discovered that, during infection, IL-17D controls the magnitude of CD8 T cell responses in part by suppressing DCs.

The pathogen-resistant phenotype of IL-17D-deficient mice is intriguing as it contradicts the prevalent notion that IL-17 family members play protective roles of host defense. Mechanistically, IL-17A has been linked to promoting neutrophil recruitment, while IL-25 promotes the recruitment of eosinophils, ILC2, and Th2 cells, thus leading to the promotion of host defenses during diverse arrays of pathogen challenge. However, the mechanisms by which the IL-17 family cytokines support protective immunity against many pathogens are not fully understood. IL-17A and IL-17C are known to induce host defense genes from epithelial cells (7, 19). IL-17A has been shown to directly stimulate DC activity, thus promoting protective immunity in *Listeria* infection (20). A previous report indicated that IL-17D activates endothelial cells. CCL2 production by endothelial cells, upon treatment with IL-17D, led to the recruitment of natural killer cells (12). In our study, during influenza A virus infection, *Il17d*^−/−^ mice exhibited a similar level of natural killer cells as WT mice. Also, our study contradicts previous reports describing the enhanced degree of infection in mice harboring IL-17D deficiency (13, 21). Since the infectious agents (vaccinia virus and murine cytomegalovirus) and route of infection (scarification and intraperitoneal injection) are different from those of our experimental models (listeria by intravenous route and flu by intranasal route), IL-17D may have differential role depending on these variances. This discrepancy suggests IL-17D-mediated cellular effects may be diverse depending on the pathogens and the nature of inflammation.

To substantiate our findings on reduced pathogen burden in IL-17D-deficient mice, we examined cellular mediators that promote bacterial clearance in the absence of IL-17D and found that CD8 T cell activity was enhanced in IL-17D-deficient mice in *Listeria* infection. This cellular phenotype was replicated in influenza A virus infected mice as well as in an ectopic tumor model, which is largely dependent on CD8 T cell activity. Notably, in the absence of adaptive immune cells, IL-17D deficiency no longer provided its protective immunity after infection, indicating that IL-17D compromises its anti-bacterial and viral immunity by suppressing adaptive immunity. Since we did not find a direct effect of IL-17D on CD8 T cell activity, we reasoned that innate mediators that influence CD8 T cell activity may be regulated by IL-17D. In a RAG-deficient background, IL-17D-deficient mice showed an increase in both the number and activated status of DCs compared with that in WT mice after *listeria* infection.

IL-17D treatment, in a co-culture of DCs from infected animals and CD8 T cells, led to CD8 T cell suppression and reduced IFNγ production. Although we did not evaluate the DC phenotype comprehensively, we found enhanced B7.1 expression, along with increased IL-6 and IL-12 production in IL-17D-deficient animals. However, it is possible that cellular mechanisms other than DCs/CD8 T cells mediate the role of IL-17D compromising host defense during infection.

To further understand the biological context of IL-17D, we evaluated the expression of IL-17D. Studies have shown that the mRNA encoding IL-17D is widely distributed in human peripheral tissues (22). Expression arrays have shown that IL-17D is suppressed in human psoriasis samples and several types of human tumors (12, 23). On the other hand, vaccina virus or mouse cytomegalovirus infection induced IL-17D expression in primary fibroblasts (13). In addition to non-immune cells, peritoneal cavity cells, including mostly macrophages and B cells, upon mouse cytomegalovirus infection expressed an increased level of IL-17D. This suggests that immune cells are a potential cellular source of IL-17D as well (21). Nrf2, a sensor of oxidative stress, was identified as a critical inducer of IL-17D expression (13, 21). Our results using bone marrow chimera indicated that non-hematopoietic compartments of IL-17D contributed to pathogenicity during *Listeria* infection and lymphoma challenge. We observed that IL-17D expression was reduced in the infected liver tissues, CD45 negative cells and hepatocytes after *Listeria* infection. However, the stimuli for IL-17D suppression were not identified in our study. It is possible that IL-17D expression is actively regulated in multiple cell types during disease development. For instance, reduced expression of IL-17D in some of pathologic tissues could be due to changes in the ratio of particular cell types. Fibroblasts in psoriatic skin or non-proliferative cells in cancer tissues are often underrepresented at the bulk tissue level. Immunostaining or cell-type specific fractionation of pathologic tissues to examine IL-17D expression might help understand the cellular sources of IL-17D.

In summary, our findings demonstrate that IL-17D regulates protective immunity against infections. In addition, we provide insight into the mechanism by which IL-17D inhibits adaptive cytotoxic T lymphocytes responses. This property of IL-17D suppressing DC activity, leading to CD8 T cell suppression, distinguishes IL-17D from other IL-17 family cytokines that are host protective during infection. Our study showed that IL-17D shares homology with IL-17 family members but is functionally distinctive in immunity. IL-17 family members utilize the same cytokine receptor chain, IL-17RA, and the same adaptor molecule, Act1. This observation raises concerns for IL-17RA-blocking antibody therapy, which potentially blocks all IL-17 cytokines without discrimination, even though some of them may have opposing roles in certain pathologic conditions. Our study provides a further rationale for understanding each cytokine in its own disease-specific setting and suggests that the therapeutic blockage of IL-17D may improve host defense during infection.

## Ethics Statement

This study was carried out in accordance with the recommendations of the University of Texas MD Anderson Cancer Center (MDACC) Institutional Biosafety Committee. The animal protocol was approved by the MDACC Institutional Animal Care and Use Committee (IACUC).

## Author Contributions

YL and SC contributed design of the study. YL acquired and analyzed most of the data. JC, CY, and SC participated in specific experiments. SC wrote the manuscript. YL and JC contributed to manuscript revision. All authors read and approved the submitted version.

### Conflict of Interest Statement

The authors declare that the research was conducted in the absence of any commercial or financial relationships that could be construed as a potential conflict of interest.

## Supplementary Material

Supplementary Figure 1 Measurement of inflammatory molecules after LPS-induced endotoxin shock. Mice were injected intraperitoneally with 5 mg/kg of LPS. After LPS injection, survival was monitored and serums were collected at the indicated time points. The concentrations of IL-6, CXCL-1, TNFα, IFNγ in serum from WT mice (*n* = 6) or *Il17d*^−/−^ mice (*n* = 5) were measured by ELISA.

Supplementary Figure 2 Bacterial burden at an early time point and challenge of listeria strain without OVA. **(A)** Bacterial burden at an early time point was similar between WT and *Il17d*^−/−^ Mice. WT (*n* = 5) or *Il17d*^−/−^ mice (*n* = 4) were intravenously infected with 1 × 10^4^ LM-OVA on day 0, and bacterial burden was analyzed in the spleen and liver on day 3. Data are from two experiments. **(B)** Bacterial burden was reduced in *Il17d*^−/−^ mice after infection with *L. monocytogenes* strain without OVA. Ten weeks old WT (*n* = 4) or *Il17d*^−/−^ mice (*n* = 3) were infected with 1 × 10^5^ *L. monocytogenes* ATCC strain 13932. Mice were euthanized on day 7 and bacteria burdens in the infected livers were measured. **(C)** *Il17d*^−/−^ mice had increased survival following *L. monocytogenes* ATCC strain 13932 infection. Six weeks old WT and *Il17d*^−/−^ mice were infected with 1 × 10^5^ *L. monocytogenes* and monitored for survival (*n* = 9 for WT mice; *n* = 8 for *Il17d*^−/−^ mice). *p* = 0.016, log-rank test.

Supplementary Figure 3 Weight loss during influenza A virus infection was similar between WT and *Il17d*^−/−^mice in a RAG-deficient background. **(A)** *Rag1*^−/−^ × WT and *Rag1*^−/−^ × *Il17d*^−/−^ mice were intranasal administrated with 13 PFUs of PR8 influenza A virus, and their body weights were measured every days. **(B)** Lung viral load was assessed via qPCR of influenza A HA RNA. Data are from two times experiment.

## Abbreviations

CFSE: Carboxyfluorescein succinimidyl ester
IL-17D: Interleukin 17D
DCs: dendritic cells
*Il17d*^−/−^: IL-17D homozygous knockout
WT: wild-type
LM-OVA: Listeria monocytogenes-expressing ovalbumin
CFUs: colony-forming units
BAL: bronchoalveolar lavage
RAG-1: recombinase-activating gene-1
HI-IL-17D: Heat-inactivated IL-17D
BM: bone marrow.
