# Supplementary figures and images for: Interleukin-17D Promotes Pathogenicity During Infection by Suppressing CD8 T Cell Activity
Source: Front Immunol. 2019 Jun 6;10:1172. doi: 10.3389/fimmu.2019.01172 (PMC6562898; doi:10.3389/fimmu.2019.01172)

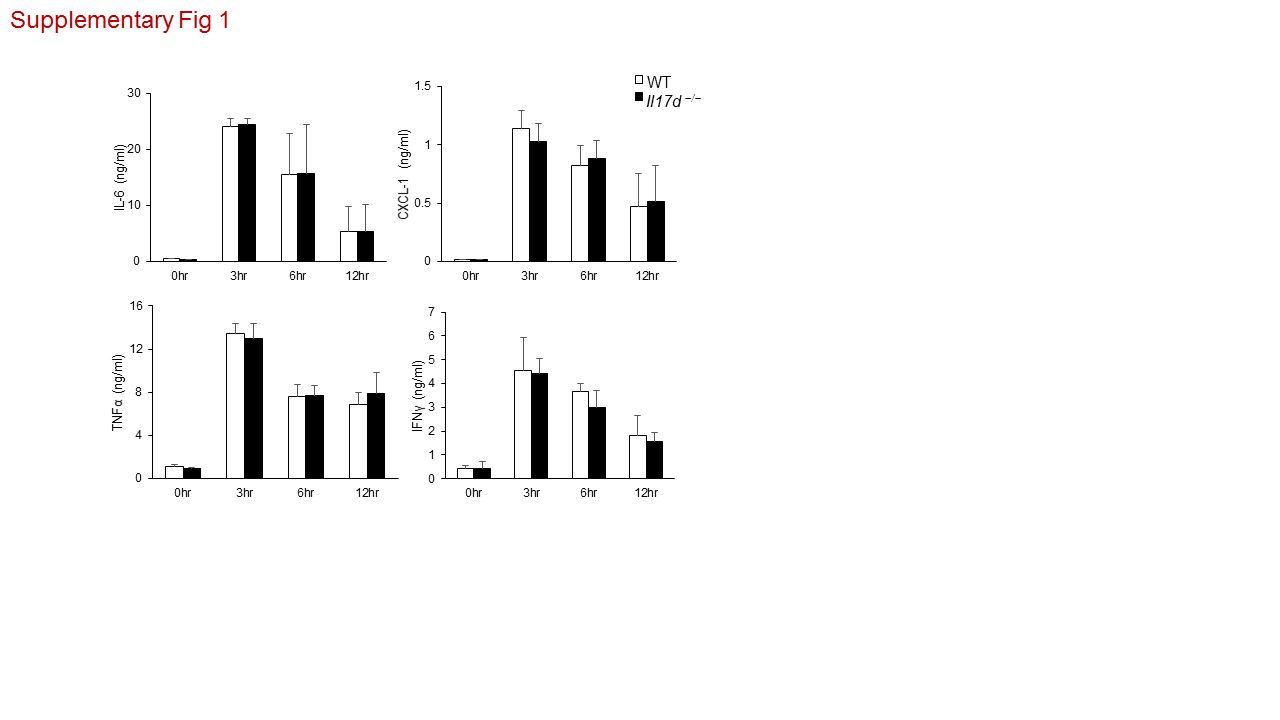

Supplement: Supplementary Figure 1 — Measurement of inflammatory molecules after LPS-induced endotoxin shock. Mice were injected intraperitoneally with 5 mg/kg of LPS. After LPS injection, survival was monitored and serums were collected at the indicated time points. The concentrations of IL-6, CXCL-1, TNFα, IFNγ in serum from WT mice (n = 6) or Il17d−/− mice (n = 5) were measured by ELISA. [file Image_1.TIF]

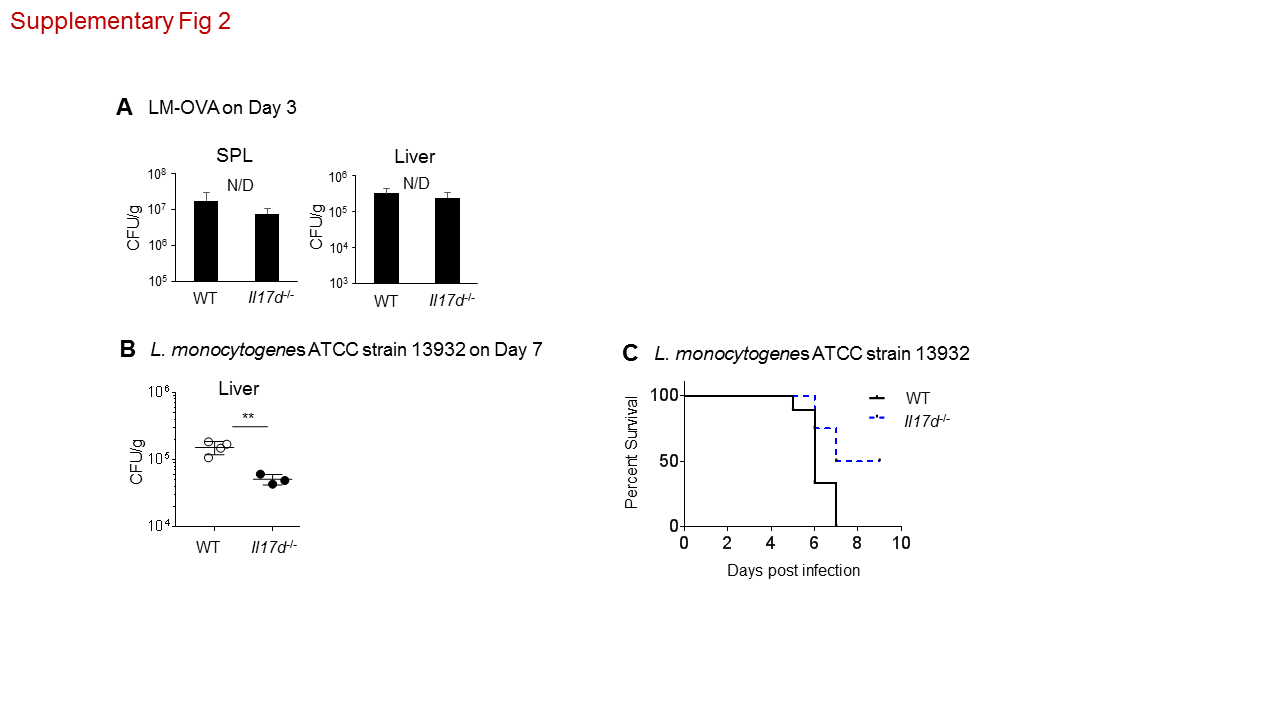

Supplement: Supplementary Figure 2 — Bacterial burden at an early time point and challenge of listeria strain without OVA. (A) Bacterial burden at an early time point was similar between WT and Il17d−/− Mice. WT (n = 5) or Il17d−/− mice (n = 4) were intravenously infected with 1 × 10^4^ LM-OVA on day 0, and bacterial burden was analyzed in the spleen and liver on day 3. Data are from two experiments. (B) Bacterial burden was reduced in Il17d−/− mice after infection with L. monocytogenes strain without OVA. Ten weeks old WT (n = 4) or Il17d−/− mice (n = 3) were infected with 1 × 10^5^ L. monocytogenes ATCC strain 13932. Mice were euthanized on day 7 and bacteria burdens in the infected livers were measured. (C) Il17d−/− mice had increased survival following L. monocytogenes ATCC strain 13932 infection. Six weeks old WT and Il17d−/− mice were infected with 1 × 10^5^ L. monocytogenes and monitored for survival (n = 9 for WT mice; n = 8 for Il17d−/− mice). p = 0.016, log-rank test. [file Image_2.TIF]

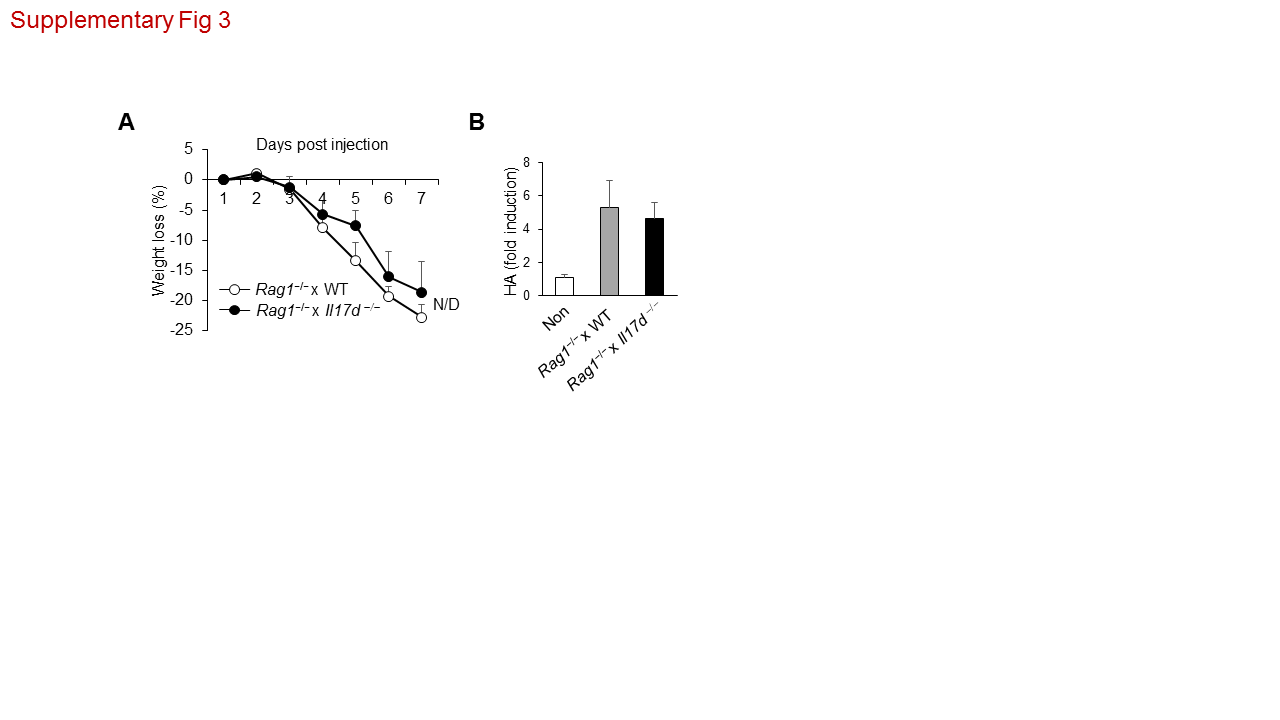

Supplement: Supplementary Figure 3 — Weight loss during influenza A virus infection was similar between WT and Il17d−/−mice in a RAG-deficient background. (A) Rag1−/− × WT and Rag1−/− × Il17d−/− mice were intranasal administrated with 13 PFUs of PR8 influenza A virus, and their body weights were measured every days. (B) Lung viral load was assessed via qPCR of influenza A HA RNA. Data are from two times experiment. [file Image_3.TIF]
